# Do Social Connections and Digital Technologies Act as Social Cure During COVID-19?

**DOI:** 10.3389/fpsyg.2021.634621

**Published:** 2021-04-01

**Authors:** Vijyendra Pandey, Arora Astha, Neelam Mishra, Rajgopal Greeshma, Govindappa Lakshmana, Sundaramoorthy Jeyavel, Eslavath Rajkumar, G Prabhu

**Affiliations:** ^1^Central University of Karnataka, Gulbarga, India; ^2^Gulbarga University, Gulbarga, India

**Keywords:** belongingness, social empathy, undesired behaviors, altruistic volunteers, social support

## Abstract

Although COVID-19 pandemic has re-orientated humans to be more physically healthy and hygienic, it has also persuaded humans to create affiliations and experience a sense of belongingness through social networks and digital technologies. However, amidst these changes, experiences of COVID-19 patients and their perception of the outside world's attitudes toward them appears to be less attended in literature which formed the basis for the current study's objectives. Using qualitative methodology, the present study explored the experiences, perceptions and attitudes of patients and their care-givers' toward COVID-19. The thematic analysis emerged with four major themes. Psychological Experiences of People was generated prominently with sub-themes indicating the perceived experiences like fear of spreading diseases to others, and the need for psychological counseling. Attitude of others toward patients and caregivers revealed that family members and relatives played a major positive role on the patient's mental health, however, the neighbor's stigmatized attitude led to several undesired behaviors. Social Connectedness was another major theme derived from the study. Altruistic volunteers, a sub-theme of Social connectedness have indicated that amidst these negative factors, one can spread social harmony by motivating and supporting the victims with basic needs, financial support, hope and social empathy. Opinions of participants for digital technology through technological aids and preventive measures emphasized an overall positive attitude as it helped the society, in general to maintain social connections as well to curb the rate of COVID-19 cases.

## Introduction

The new respiratory disease COVID-19 is highly contagious disease. The rate of its spread is much higher than the previous biological pandemics (Varshney et al., 2020). In order to prevent its outspread, ruling authorities across the world have announced mandatory home quarantine as a preventive measure. To minimize its spread a temporary shutdown has been imposed nationally in different ways, i.e., closing down of academic institutions, ban in public meetings, restrictions in social gatherings and social distancing. On contrary to the basic instinct of human kind, people were obliged to stay physically disconnected. While following these preventive measures, reports indicate the use of digital apps in connecting people worldwide and providing knowledge to be healthy (Iqbal, 2020). Being in home quarantine and isolation can take a toll on both the mental and physical health. Various negative feelings such as anxiety, depression have been noticed among people during the lockdown period due to the pandemic (World Health Organization, 2020a). Along with this, numerous literatures have emphasized on the psychological effects experienced by individuals during previous pandemic eruptions like Ebola, MERS-Cov, SARS, and H1N1 (Sun et al., 2020). Besides, studies have statistically provided evidence of older patients' co-morbidity of Ebola with anxiety, depression, and post-traumatic stress disorder to be 24.9, 47.2, and 21%, respectively (Rojek et al., 2017). Notably, even the caregivers of these patients had been diagnosed with depression and anxiety; in fact, about 45% of them opted for psychological counseling albeit at a later date (Smith et al., 2017).

On the brighter side, few studies reported that acts of social wellness, such as community support, positive interactions with patients and empathy have led to positive psychological well-being of patients in such crisis (Kama et al., 2017). Studies like this have illustrated the role of social connectedness to ease psychological impacts. During COVID-19, due to imposed restrictions, modern technology could be the only way to maintain social relations. Thus, this study tries to examine the role of social connectedness in social cure.

### Social Connectedness

The feeling of connectedness is the basic need of human beings. During this pandemic, it has been reinstated since the time people were advised to follow “social distancing” norms even within the family. Recent researchers have argued to change the terminology of “social distancing” to “physical distancing,” which would effectively reflect the actual meaning of the preventive measures to reduce the feeling of social disconnection (Sanderson et al., 2020). Nevertheless, the foremost drawback through the norm of “social distancing” that has been observed is that people at large began to develop an apathy to “social wellness behavior,” whereby they began to discriminate COVID-19 patients, their family members, and at times even the “caregivers/care providers,” i.e., doctors, medical personnel, police officers among others (Graupensperger et al., 2020). However, if we were to question ourselves on what is the phenomenon of “social wellnesses!” In order to understand the same, one needs to understand the experiences of patients and the attribution of the term “social connectedness.” Rossi et al. (2012) defined it as “a person's subjective awareness of being in close relationship with the social world.” Notably, this attribution of self reflects cognitions associated with an individual's on-going interpersonal closeness with the social world. Therefore, “social wellness” may refer to the way a person develops social health, while expanding connection with others in the dwelling/society. It alludes to a person interacting in a positive way, while creating and maintaining healthy relationships, which in essence serve a meaningful purpose in life. Thus, both social health and social wellness become an essential dimension for emotional, psychological as well as physical health (Kingsep, 2019).

Research in the past have shown the positive correlation of “social connectedness” with emotional resilience, conflict management, life satisfaction, and self–esteem (Fraser and Pakenham, 2009; Stavrova and Luhmann, 2016). Richards (2016) stated that despite an increase in income, the participants of his study were found to be happier when their social connections grew positively, which effectively meant that people did value social connections more as opposed to “income.” On the contrary, chronically lonely people showed negative health outcomes, including the aspect of “addiction,” be it to drugs, alcohol and others. Importantly, people with low social connections proved to be more toxic than obesity, hypertension and high blood pressure (American Psychological Association, 2017; Tate, 2018).

Interestingly, it was found that people who maintain healthy social connections could actually produce more antibodies through their immune systems, which eventually help them to fight diseases and provide longevity to the body (Marchant, 2013; Pappas, 2013). Studies have also highlighted the role of mirror neurons that help in producing empathy, sympathy, compassion and a feeling of belongingness within or others, often by inducing pain in selves and/or others. This goes on to prove that the aspect of “empathy” does make a person take proper moral or humane decisions eventually increasing the feeling of belongingness, while triggering an intent to help others (Lamm and Majdandzic, 2015). Psychological researchers have explained this phenomenon as reinforcement and vicarious learning (Kazemi and Rostamian, 2016; Pfefferbaum and North, 2020). Furthermore, people having good social connections were found to have better eating and exercise habits, which could be the result of reinforcing healthy habits by friends.

### Social Connectedness and Digital Technology

The “small-world problem” hypothesis showed social connections to be so dense that every person on this planet could be connected to just a few intermediaries. The experiment “Six Degrees of separation” conducted by Stanley Milgram (Milgram and Travers, 1969; Maier, 2019) found that to help a person in receiving a letter from one end of the world to the other, there would be in a need of only six hands exchange. In other words, on an average, only the involvement from six people could create nodes in establishing connections. Researchers have also shown that the “small-world network model” could be used to explain social networks, computer networks, neural networks and wireless networks (Watts and Strogatz, 1998). Sohn's (2017) “small-world network model” highlighted the difference in the number of people that could be connected indirectly through social network and a wireless network connection (Uzzi et al., 2007). Although social connectedness and belongingness has been identified as the third key need postulated by Maslow in his “hierarchy of needs,” extensive research has shown and even reiterated the importance of “connectedness” to maintain a good physical as well as mental health (Baumeister and Leary, 1995).

Bronfenbrenner (1994) also discussed the interpersonal interactions of human at different levels that include micro, meso, exo-, and macro systems. With modern technology, interactions between these spheres have become more compatible (Ashiabi and O'Neal, 2015).

The different palette of communication tools such as email, text messaging, WhatsApp, Facebook, etc. have all provided a platform to interact across the globe, albeit in a virtual mode. A few researchers divide social connectedness into two prominent divisions: (a) one to one connectedness (through text messaging) and (b) large basis of connectedness (through Facebook or Instagram) (Bel et al., 2009). Both one to one and large basis connectedness do help individuals to “connect” with family, friends, and the world at large.

Since the outbreak, Social media has been over-embellished with its persisting and exclusive coverage on the pandemic. For instance, the News18's (2020) strange news of two women doctors being threatened and abused, and asked to leave the grocery shop and empty their flat on an immediate basis by their neighbors had gone viral in social media. On contrary, India TV on 23rd April 2020 reported certain pleasant incidents of the warm welcome of recovered patients by the neighbors (India Tv, 2020). Interestingly, the *Washington Post*, on 8th April (2020) in one of its news bulletins, opined that such contrary behaviors have been observed across the world (The Washington Post, 2020). Social media has been a disseminating tool for both information and news, including many fake ones, ranging from the recovery rate, death rate, and vaccine development. World Health Organization team has stated, “The 2019-nCoV outbreak and response has been accompanied by a massive ‘infodemic,’ some accurate and some not, that makes it hard for people to find trustworthy sources and reliable guidance when they need it” (World Health Organization, 2020b). The term “infodemic” was meant to allude to an over-abundance of news. During this pandemic, World Health Organization (2020b) prompt response about infodemic calls for one's immediate attention to cross check the reliability of incidents that happen in the community.

### COVID-19 and Digital Technology

Scientists and researchers have used technology, ranging from high-tech robots to low-tech masks, to the best, to fight this pandemic. Robots have been used massively for cleaning, sterilizing, emitting Ultra Violet (UV) rays and delivering food to patients or the needy during quarantine in order to limit human-to-human contact. Digital technology has also been harnessed to provide innovative ideas; e.g., when patients were short of ventilators, researchers and engineers have formed several online groups on platforms such as Telegram, Facebook, Instagram etc. to share knowledge on manufacturing ventilators. This apart, Artificial Intelligence (AI) was also used; in fact, Blue-Dot software had triggered an alert even before WHO on this pandemic by analyzing data provided by the different agencies (Steig, 2020). The usage of AI for image-based analysis in Computerized Tomography-scan (CT-scan), understanding the working of coronavirus to produce drugs, telehealth has been an immediate relief provided by the technology.

Although largely technology has played an uplifting role, it has its own hazards; for instance, the aspect of individual privacy. When the world was facing global lockdowns, various countries using technologies such as AI tried to track down its people. Even though this was intended for a “noble” cause, it is also disadvantageous, as it leads to intrusion upon an individual's privacy (Tétrault, 2020). Media coverage, especially through television has also worked bi-directionally. While this pandemic continues to cause harm to society, scientists and researchers are no longer bound by geographies due to technology-based support. The technological advancements has helped immensely to provide different COVID-19 testing aids and treatment facilities across the globe within a short span of time.

### Social Connectedness and COVID-19

Following preventive measures and use of digital apps help to connect people worldwide and providing knowledge to be healthy (Iqbal, 2020). Being in home quarantine and isolation can take a toll on both the mental and physical health (Altschul, 2020). The role of social connectedness cannot be ignored during the pandemic.

Thus this study aims to record insights from the patients and caregivers' perception and experiences toward the COVID-19 as well as the usage of technological aids to maintain social connections in the unforeseen and disastrous situations such as this pandemic. In addition, this study explores attitudinal differences of people toward COVID-19 patients. The salience of this study is to explore and attempt to understand how social media network and social connectedness aided through digital technology play an important role in maintaining relationships as well as to see if they act as a social cure during the pandemic.

## Methods

### Design and Participants

This study used an exploratory research design to understand the proposed objective. The participants of this study were chosen, using a purposive sampling method. Before conducting the interviews, informed consent was taken, and confidentiality was assured. This study included 38 adults (Male = 21, Females = 17) across India, between the age group of 21 to 40 years (Mean = 28.5, and SD= 4.5) having at least graduate-level qualification. Out of 38 participants, 20 were COVID-19 recovered patients, while 18 were caregivers.

### Procedure

This qualitative study aims at exploring the first-hand experiences of the recovered COVID-19 patients, along with their caregivers during the pandemic. Telephonic interviews with the participants were recorded after getting their due consent. They were also assured of total confidentiality, and were told that the data would exclusively be used for research. On an average, each interview lasted about 40–45 min. A set of semi-structured questions were prepared to conduct the interviews, in consultation with the experts. Semi-structured interview questions were framed after several scrutiny of experts. To ensure the inclusion and exclusion criteria few questions were asked initially during telephonic interview. For instance, a few questions like following were asked: (1) During the crisis of COVID-19, what was your experience of social distancing? (2) Has it affected your emotional well-being? and (3) What were your strategies to cope up with the same?

Few questions asked to COVID-19 patients: (1) What was your first reaction the moment you got positive report for COVID-19? (2) Who all has helped you to fight this situation? How did they help you? and (3) What was the perception of others toward you once you recovered from such illness?

Few questions asked to caregivers: (1) What was your experience and approach to help the person who was diagnosed positive for COVID-19? (2) Except being COVID-19 as a contagious disease, what all could be the reason for not helping needy?

### Data Analysis

The data of this study was analyzed using Braun and Clarke's (2006) Thematic Analysis research method. Thematic analysis is a method for identifying, analyzing, organizing, describing and reporting themes within a data set. The approach of thematic analysis suits to explore and identify prominent themes of the data collected here. After each telephonic interview, recorded content was carefully converted into verbatim and thoroughly reviewed by the researchers. This was done to assure the accuracy of the information provided by the participant transferred to the paper. During the analysis of the data, researchers have gone through verbatim again and again to refine the understanding of data and identifying the words, sentences which were providing similar meaning. Further, responses provided by the participants were noted under different columns. Each column represented the responses which provided similar meaning and through literature review suitable sub-themes and themes were assigned. The criteria provided by Guba and Lincoln (2011) for evaluation of qualitative research was taken under knowledge during analysis. Through data analysis each criterion, firstly credibility of information was assured through repeated reviews of data gathered and transferred in verbatim. Secondly, transferability of knowledge via mentioning actual responses of participants, their characteristics was assured, which could help other researchers to generalize their findings in respective findings. The proper division of step-by-step administration by researchers assures the non-biasing and removes dependability on limited researchers. Lastly, the fourth criterion of conformability was assured when the final result of the data analyzed and the accompanying process involved was reviewed by researchers.

## Results and Discussion

The present situation of COVID-19 has brought diverse experiences to the human kind. This outbreak has led people to follow various preventive measures such as home quarantine them physically distanced from each other. At the same time, technology is on the peak which smoothen the route to connect with each other which may have positive as well as negative impacts.

In this research, data was collected from 38 participants, in which 20 were COVID-19 recovered patients, while 18 were caregivers. Upon employing thematic analysis, four major themes, along with 10 sub-themes emerged. They include Psychological Experiences, Attitude toward the patients. The description of these four themes and overall, 10 sub-themes are as follows.

### Psychological Experiences of People

COVID-19 has affected people both physically and psychologically. Participants have reported various psychological struggles that they experienced on the basis of which three sub-themes evolved such as: *perceived experiences, concern toward patients and the negligence of psychological counseling*. The significant findings from the experiences that have been observed in the obtained data of this study are mentioned below.

### Perceived Experiences

Participants, who recovered from COVID-19, as well as their caregivers had gone through the distressing psychological experiences during the critical time of their illness. This primarily included depression and anxiety, while some were stressed, and a few others traumatized. However, the causal factors for the disturbances between these two groups were found to be different. Herein, it is vital to note that these experiences were also the result of social distancing, home quarantine and unexpected financial crisis faced by the participants.

However, the overall responses under this sub-theme of “*perceived experiences*” were majorly negative because people were afraid of this uncertain situation. This has been also observed in previous epidemics in history (Smith et al., 2017; Varshney et al., 2020).

### Unpleasant Experiences of Patients and Caregivers

Among several psychological distress faced by the participants, anxiety was the most dominant, followed by depression, stress and insomnia. Rehman et al. (2020) reported a wide range of psychological experiences, such as the perception of risk, fear, anxiety, stress, and depression level among the different populous, caused by COVID-19. A male participant AK for instance, encountered a lot of anxiety during his self-isolation. He stated “*one day... I heard from the cabin man that. my neighbors patient suffering from breathlessness and sent to a different ward. And it created tremendous anxiety in me. I had chosen a single cabin room because I don't want to see the others' packed body*.”

While a few reported their experiences of anxiety, others shared their reactions to various traumatic incidents. For example, a female participant DK stated, “*I was so much traumatized that everyone came to know about my reports. I felt like I can't do anything. It was so depressing*.”

The anxiety level was higher among healthcare professionals and women during pandemic events (Hacimusalar et al., 2020). However, in the present study, such gender-based differences could not be found. Few participants, irrespective of them being patients or caregivers, experienced higher stress levels. For example, a male participant NJ stated, “*Me, my wife and my youngest daughter were tested positive for Covid-19. The day before the report came; I had cremated my mother…. Due to this lockdown…caused me a lot of financial crisis. And the report of my daughter has shaken me inside and made the situation more stressful*.” In certain studies, the anxiety level was high among the caregiver community (Cici and Yilmazel, 2020).

Caregivers in specific have reported insomnia symptoms. A caregiver SD stated “*I'll get up at two o'clock/three o'clock…. she was in a separate room…. I used to peek from the window itself and come back. Like the Attention, apprehension what we'll happen? will she recover or not*?”

Some of the caregivers claimed that the disturbances were related to the uncertainty of future and fear of loss of their family members.

These psychological disturbances led the participants to develop insomniac conditions too. Caregivers in specific have reported insomnia symptoms. A caregiver SD stated “*I'll get up at two o'clock/three o'clock…. she was in a separate room…. I used to peek from the window itself and come back. Like the Attention, apprehension what we'll happen? will she recover or not*?” The caregivers claimed that the disturbances were related to the uncertainty of future and fear of loss of their family members.

This category therefore, revealed that both patients and caregivers experienced anxiety, depression, stress, and insomnia due to various issues. However, the causes of experiences differed in both groups. While patients' psychological disturbances were mostly concerned with isolation and tensed panic environment, caregivers' anxiety was more associated with an uncertain future, and the well-being of their loved ones due to lack of proper treatment.

### Pleasant Experiences of Patients and Caregivers

Despite negative psychological experiences, a few people found home quarantine and lockdown to be pleasurable in the initial phase, as they were having quality time. This sub-theme emphasizes on the positive experiences of the participants. A female participant NJ stated, “*my mom cooked a lot so I was so happy. I am a very homey type of person.... stay with my parents. So, it was very fortunate.... I enjoyed talking and spending good time with family. Also, I wasn't living in my house for few years and I felt that I need to spend time*.” Concisely, the participants enjoyed being at home and spending quality time with their families.

Hacimusalar et al. (2020) noted that the anxiety level was higher among healthcare professionals and women. However, in the present study, such gender-based differences could not be found. Furthermore, findings similar to this study were also noted by Cici and Yilmazel (2020) which stated that the anxiety level was high among caregivers.

However, the overall responses under this sub-theme of “*perceived experiences*” were majorly negative because people were afraid of this uncertain situation. This has been also observed in previous epidemics in history (Smith et al., 2017; Varshney et al., 2020).

### Concern Experienced by Patients (Causing Others)

While interviewing patients, most of them were concerned more about their family members toward COVID-19. Fear for their significant partners and children were more prominent. For instance, a female participant NY stated “*my husband did not have any fear of getting corona we had some fear for children... because if they got... then how to take care of them we can't go near them*.”

These findings reflect the phenomenon of higher risk perception (Cowling et al., 2010) that is during the pandemic, people perceive more risk of spreading infection. This increases the motivation to protect oneself and others to avoid infection. Notably, Simione and Gnagnarella (2020) also found that higher the risk perception in a given group of respondents, the higher is their concern toward health. Perceived severity and self-efficacy have been found to be positively correlated with self-isolation in order to prevent spreading of COVID-19 (Chen et al., 2020).

### Negligence of Psychological Counseling

This sub-theme focuses mainly on the lack of psychological counseling services that participants thought was necessary. The Indian government-initiated state-specific intervention strategies, tele-psychiatry consultations, toll-free numbers specific for addressing the psychological and behavioral issues faced by the COVID-19 victims (Roy et al., 2020). However, efficacy of these psychological counseling on patients and caregivers has still been unreported and is thereby ambiguous. A male participant PG stated, “*Apart from taking medicines there should be a program*s *like psychological counseling, which the govt or anyone can arrange so that they can help covid patients from psychological issues they are facing*.” Another caregiver participant, AB stated, “*As I say that it is more on the mental side, not on the physical side*.” In fact, some of the caregivers went on to highlight that although they did not contract COVID-19, their mental health was as affected as of the patients. While patients were being treated by the hospitals, there was no psychological support provided from the government for the caregivers. Das (2020) emphasized the need for psychological interventions at a large scale to counter the after-effects of post-traumatic stress, frustration, stigmatization etc. Thus, there is indeed a need to have a strong policy for such provisions.

### Attitude of Others Toward the Patients and Caregivers

This main theme, which originated based on shared experiences, sheds light on attitudes of family members and support from neighbors as well as peers. This theme has been sub-divided into two, i.e., during recovery and post-recovery, which in essence, shows the differences in people's attitudes toward participants both during and after recovery. This theme also involves some pleasant as well as unpleasant experiences, which are stated as below.

#### During Recovery

This was a time when patients were asked to isolate themselves, and avoid making contact with family members and others. The differences in the experience of social connectedness have been due to their varied situations, such as when the participant was infected by COVID-19 vis a vis after him/her getting cured. Although family members were concerned about patients, another dominant social support system, i.e., the “neighborhood” was found to be failing in establishing and maintaining social connections with the patients.

##### Family Care

In the initial stages, parents have had difficulty in accepting their son and/or daughter diagnosed with COVID-19. However, family members reported that they gradually provided the required support to the patients with proper care. A male participant AK stated “*they all were just motivating us, saying that no problem, it happened just happened, be positive*.”

Though family members and near ones initially displayed denial and non-acceptance, eventually they accepted and found the courage to support their loved ones, which increased their feeling of togetherness.

##### Societal Attitude

This sub-theme highlights the discriminating, stigmatizing and traumatic treatments by the neighbors toward the patient and caregivers. World Health Organization (2020) stated that having more ambiguity due to a new disease may lead to unconscious discrimination, which reflects through stigma and stereotype. A male participant PG stated “*They ignored me.....my name was given to the newspaper and people announced it in the whole colony. People used to run away from our house as if there is a ghost inside. Everyone got to know*.” A few statements highlighted the discrimination and stigmatization of the disease, resulting in name-calling such as “virus home.”

Contrary to these negative attitudes, a few interviewees indicated a positive attitude by their neighbors also. A female participant AA noted, “*One of our neighbors is so good. They used to come home only in night, till then they took care of children. We didn't feel the need for friends; our neighbors helped us thoroughly*.”

Since caregivers were directly or indirectly in touch with the patients, the stigma toward patents might have been generalized toward caregivers as well. Overall, the neighbors' attitudes toward the patients as well as the caregivers have been negative. The behavior shown could also be understood through negative dominance theory (Covello et al., 2001; Glik, 2007), which states that when people are fearful, they give more weight to negative information and respond accordingly.

##### Peer Support

Support provided by the friends and colleagues has worked as a healing system to many survivors. It has been noticed that friends of the patients have helped their family members too and assured their presence in an emergency. A female participant MB noted “*They were being too supportive ….actually my mum was Home alone. my friends... used to bring all the fruits and required materials by ordering it online and it would be delivered at home. So that was a good thing*.” A positive social connectedness and concern by the friends stayed prevalent during as well as after the recovery period.

#### After Recovery

This second sub-theme focuses on the attitude and perception of patients after their recovery. In addition, caregivers reported that a few neighbors initially avoided them due to risk perception but, such behaviors subsided eventually.

A male participant AK responded, “*Since, no one in my area had it before me, I had become like the godfather for everyone. Whoever got it later called me for suggestions*.” Similar differences in attitudes were observed by caregivers, as others sought help to know the ways to maintain hygiene and prevent contracting COVID-19 from patients. This showed that people sought help from both caregivers and patients but for different reasons. This could be explained with the “mental noise theory” (Baron et al., 2000), which explains that people during a pandemic, get stressed, and they attend to a great deal of internal “mental noise,” albeit unconsciously, which in turn results in a lesser ability to attend to externally generated information. Notably, this indicates the change of behavior in people in which, negativity toward someone is not intentional or fabricated, but rather a representation of chaos and fear.

### Digital Technology

This main theme highlights the dual role played by the technology. The digital technology has worked as a carrier to spread fear as well as knowledge about the disease condition and at the same time extent support to the community.

In its sub theme, at one place social media is working as a carrier to spread fear causing anxiety, depression. On other hand through ease in communication through digital technology has worked wonders to provide support and strength to the society.

### Influence of Digital Media

This main theme emphasizes the influence media has on the individual's thoughts, perceptions and behaviors.. Gao et al. (2020) found that 80% of the participants who were exposed to social media had a prevalence of depression and anxiety. Das (2020) provided evidence that social media news has been the leading cause of over-reactive behavior, especially of the Indian people. With technological advancement, there are various platforms, which provide information to the viewer. Some platforms are very recent and some have been there for a long time.

The experience of listening to unpleasant news related to COVID-19 significantly influenced the people. For instance, a male participant LD stated “*In the initial times.... hearing all such news that is many people died and there is no cure there is no treatment for this disease hearing all those stuffs I was getting disturb*ed *that if I will get what will happen. So later, I didn't follow them at all to avoid panicking*.”

This sub-theme showed that most of the people relied on newspapers than on news channels or any social media platform.

On the contrary, the contemporary news feed from digital media platforms such as WhatsApp, Facebook, and Twitter form to be posing as a major problem than the news from television. A female participant ST shared “*I remember someone posted a list with my name. I got mad. I said okay, you take precautions and all... but don't disclose the names of people. And this resulted in back to back comments and quarrel and I just left that group*.”

Apejoye (2015) stated that information and facts shared through the means of both newspaper and television are more credible than social media platforms. The report provided by World Health Organization (2020) regarding infodemic seems to be true in this study also. However, results of this study show more of the negative aspects of the digital media, which is an indication for the government, and respective organizations to look upon this issue seriously, as news from these sources could be channelized to help create positive influence, which would bring the community closer.

### Technology Aids: Perception and Attitude

During home quarantine and isolation, the only convenient method to stay connected was through mobile phones and social media platforms. This advancement has provided an opportunity to embrace social connectedness in a distinct way. Chen et al. (2020) mentioned about the ease provided by technological advancements during COVID-19 in order to connect with acquaintances. The perceived support provided by technological advancement worked as a mental component in recovery of patients. The advancement commencing from cell phones to the development of various tools to diagnose COVID-19, as well as to restrain the spread, has created an enormous difference in stalling the pandemic. This theme emerged because of shared experiences by the participants about these technological advancements by further focussing on social connection and social media attitude toward treatment and related aids. More or less the technology evolved as a companion who provided relief by even connecting the stranded people with their close ones.

### Opinion Toward Treatment and Related Aids

With the advancement in technology, treatment to certain diseases has been made possible to diagnose and be cured. Researchers worked day and night to provide credible diagnostic systems to COVID-19. The purpose of this cannot meet until the common man does not trust and perceive it as a cure to the disease. This sub-theme is further divided into two divisions that include the perception of people toward COVID-19 testing, and applications, which promote a healthy lifestyle.

#### Opinion Toward COVID-19 Tests

Two prominent testing, i.e., Antigen Test and RTPCR test were widely used to diagnose the presence COVID-19 virus in human body. Antigen test also known as Rapid Antigen Test—(RAT), provides results within 4–5 h, while the RTPCR test provides results after a span of 2–3 days. It was an attempt to know the patients/ caregiver's perception related to the credibility and reliability of both the tests for diagnosis.

A male participant KJ mentioned, “*what I know is if rapid test there as false negatives... as far as I have seen if it comes positive, RTPCR also comes positive around 99% cases.... So, if it is negative with symptom better to go with RTPCR.*…”

On contrary to this, a few participants mentioned the use of antigen test as the need of the hour. A few reasons mentioned by participants included the point of India's humongous populous, coupled with the virus spread being so high. They further stated that for the government, it would be more convenient to do testing through rapid tests. On other hand, experimented medicines and treatment provided by the government found to be not more reliable than the Ayurvedic *Kada*, which is a drink prepared with the use of herbs to boost immunity.. A female ST mentioned, “*I trusted more on ayurvedic treatments*.”

#### Opinion Toward Apps

This subtheme emerged in accordance with the claim done by various health-based applications to provide a good knowledge base to stay healthy (Warman, 2015). In contrast, the majority of participants denied the utility of such apps and any exercise-based application. Most of them favored ancient Indian practices to stay fit, such as practicing “*Yoga*,”*and* “*Pranayama*,” and consuming “*Kada or maintain a balanced diet*” to stay healthy and boosting immunity.

A male participant PG responded, “*I didn't prefer it that much. I used to do yoga and kappalbharti and I have continued that in lockdown also and when I was in quarantine that time also*.”

The responses provided by the participants were not in the favor of using any of the health-based applications. Husain and Spence (2015) provided evidence that a few health applications may accurately show some aspects of health, but it would not be wise to solely rely on such apps. This shows the trust and beliefs of people on traditional knowledge.

Although, in this study the advancement of technology has been found to be beneficial in some domains, the importance of traditional practices could not be overlooked. In support of this, the traditional knowledge for “*yoga*,” exercises and to prepare medicines and “*Khada*” to cure the illness has also been preferred and given the importance of traditional and indigenous knowledge.

### Social Connectedness

In order to follow social distancing and home quarantine, the only way to connect acquaintances was through social media platforms and mobile phones. In this theme, experiences of patients and caregivers are described which highlights the usage of digital media platform.

### Social connection and Social Media

This theme emerged in the viewpoint of an increase in the use of social media as a helping aid for people to connect with others. It has been observed that the order of staying at home became easier due to technological aids. A male participant for instance, reported: “*Thank god! We have this internet thing otherwise it could be difficult to stay home for so long*.”

Moore and March (2020) found that social media helps to connect people with their loved ones and bring down the feeling of being in isolation during this pandemic. Most of the recovered patients mentioned that the frequent calls by the acquaintances through social media platforms they received during the lockdowns provided a lot of strength and mental support.

When interviewees were further probed, they revealed that through social media connection and in-person meet, they got to know certain new trends being carried around them. A female participant AK for instance, stated, “*It was a new experience that we were exploring and we were doing something.... Every Saturday and Sunday, there were some Tam bola Night, some other means or some other puzzle game. So, it was very interactive*.”

None of the participants preferred in-person meeting against social media connection, and added that it was the need of the hour. Thus, interactions in social media platforms helped people to cultivate a sense of social connectedness.

### Preventive Measures

Most of the participants talked about their respective concerns, and followed preventive measures, such as staying at home in order to restrain the spread of the virus either to themselves or to others. A male participant noted, “*Home quarantine was mandatory for oneself and others*.” Other preventive measures, such as wearing masks, sanitization, and “social distancing” were acknowledged and approved by the participants. Some of them even talked about educating others to strictly follow these preventive measures. A male participant, PG stated, “*We ourselves* need to *sanitize our society*.” Thus, people took care of themselves as well as others in order to remain safe from the virus' onslaught.

### Altruistic Volunteers

This sub-theme has emerged as an additional finding to the primary objectives that were framed. Although COVID-19 has been a contagious disease, most participants have voluntarily helped others in need. The life instincts have been believed to be the cause of such self-preserving behaviors. It is believed that people due to life instinct would self- preserve themselves. Mawson (2005) explained such contrary behavior by linking it to a massive panic among people during such pandemic. On this contrary belief, literature shows that pro-social and altruistic behaviors are predominant.

A male participant, PG stated, “*Yes me and my social club members used to cook food for 200 people who were migrants. It went for 2 months. We use to prepare food for two meals. It was just a blessing*.”

Moreover, separation and guilt of not helping others is an unbearable psychological stressor than the physical danger (Glik, 2007). This may be attributed to the altruistic behavior shown by most of the participants during this pandemic. This kind of helping attitude builds the community as a whole, and provides a mental cure that strengthens the feeling of togetherness, which possibly plays the lead role in the healing process. Thus, it may be affirmed that social connectedness does enhance the sense of belongingness to the society that can sustain even during a pandemic.

**Figure 1:**
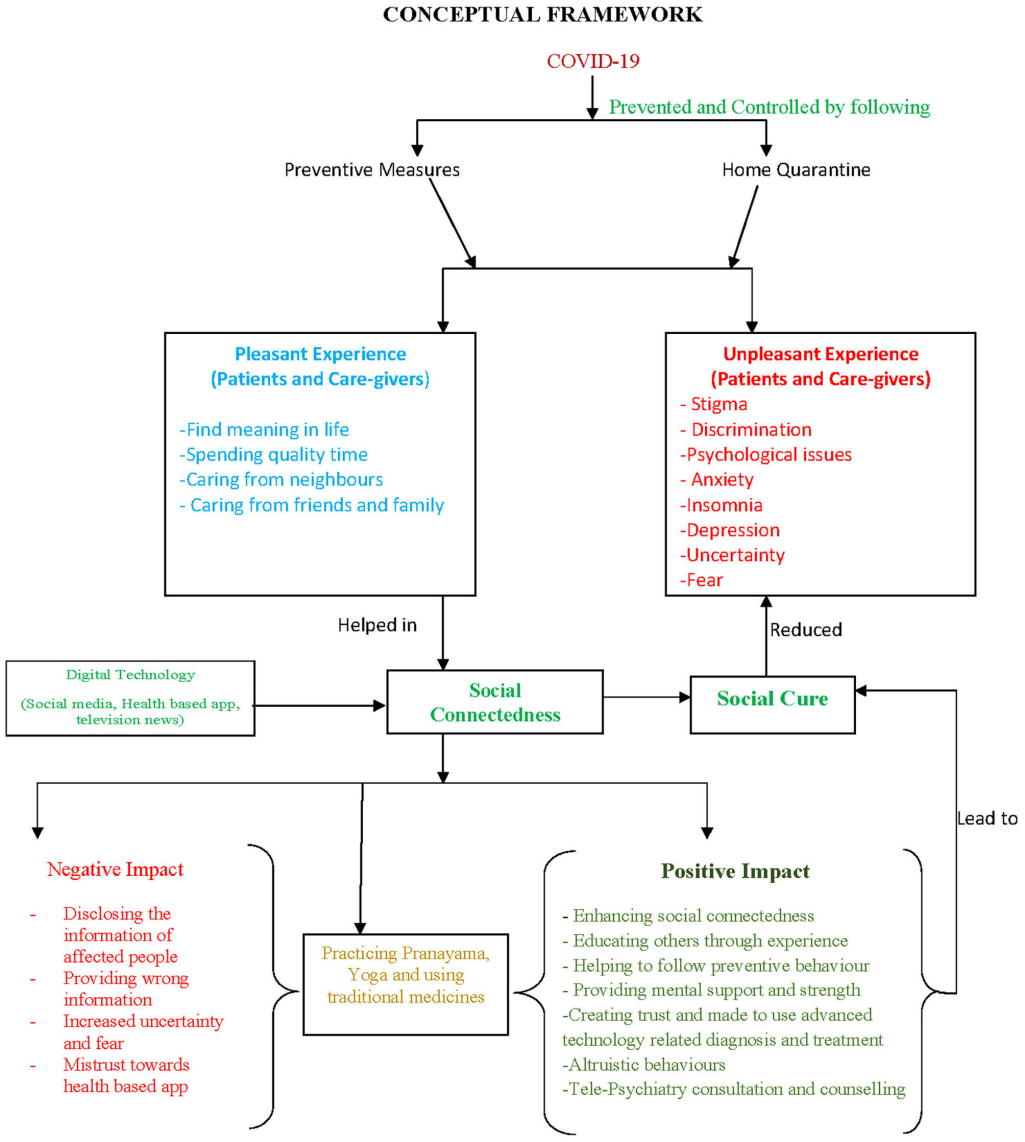
Conceptual framework derived from findings of the study.

This study reported that both patients and caregivers have experienced discrimination, stigmatization, as well as psychological disturbances during the battle against COVID-19 and find certain meaning to their lives. Conspicuously, negative experiences faced by both the caregivers and recovered patients have felt the need for psychological counseling. The changes in the attitude of neighbors, both before and after a patient's recovery has been seen clearly, indicates the necessity of socio-psychological theories and their applications. Although many caregivers did not contract the disease, they emphasized that their mental health was ignored drastically, while they were attending to the patients. Patients, caregivers and Doctors could interact more feasibly than ever in this pandemic, which alters the way to provide support and guidance to the patient and caregiver which impact psychologically (Sanderson et al., 2020). Importantly, this study also highlights the need for counseling and therapeutic services in all the government and private health sectors. Therefore, proper policies, strategies must be implemented through top-down approach. The study also provides insights regarding the role of self-introspection to be healthy, while enabling others to remain healthy and safe. On the contrary, negative influence of digital media, which highlights the need for the government to implement policies to regulate the information that the media delivers, especially social media. The conceptual framework derived from the findings of the study could be found in Figure 1. The study also provokes researchers, employees and health officials to design guidebooks and prepare oneself as well as others to create immediate awareness and knowledge among the common people for any such similar catastrophes in future.

## Conclusion

The major findings of this study highlighted the positive role of the strength provided by the social connections to the patients and caregivers. In the cases where support was not provided, although the reasons varied, both caregivers and patients did experience psychological disturbances. While patients were mostly concerned with isolation and tensed surrounding, the caregivers were anxious about their uncertain futures, financial crisis and well-being of their loved ones. Although the caregivers were not infected with the virus, they reported that they could also not be free from fear and agony due to multiple situational causes. Further, patients opined that adequate counseling services should be mandatory for the patients as well for themselves; whereas caregivers shared that, their mental health also needs greater attention. In addition, although family members and friends supported the patients, as well as caregivers, the support of the neighbors was compromised most of the time due to the fear of contacting the disease. However, after recovery, the neighbors welcomed them to the extent that they sought help regarding preventive measures.

This study also highlighted the ease to connect with loved ones through digital technology which was found to be a blessing in disguise. The voluntary help provided by the participants highlighted the concern for social welfare and shows the positive use of digital technology propounding social connectedness.

The study also revealed the way the attitude toward the COVID-19 patients is considerably different among the individuals due to certain existing stigma, varied information through social media as well as uncertainty about the prognosis of the disease and their treatment. Further, this states a dire need of proper awareness, counseling, and several other researches, which can bridge the gap between the existing practices, varied perceptions and attitude which may further help us in treatment of COVID-19 as well to enrich the theoretical, cultural as well as policy-based contributions.

## Limitations and Further Recommendation

Owing to the tensed situation created by the pandemic and reduced interaction among individuals, the researchers experienced difficulty in obtaining a varied representation of sample, e.g., socio-demographics that in essence play a major role in attitudes of individuals toward study variables. Moreover, several important variables, such as personality, religion, caste, age, comorbidities, possible financial constraints, and traditional practices to avoid the illness, variations of administration to tackle such issue and available resource on or before COVID-19 could be incorporated in future studies which may provide a cure paradigm for any such calamities in future. Integration of multiple disciplines, i.e., social science subjects with medical science and technology could be taken together to understand, as well as map the understanding of acts toward such situations in the future.

## Data Availability Statement

The raw data supporting the conclusions of this article will be made available by the authors, without undue reservation.

## Ethics Statement

The studies involving human participants were reviewed and approved by Departmental Review Board, Central University of Karnataka. The patients/participants provided their written informed consent to participate in this study.

## Author Contributions

VP, AA, NM, and RG conceived and performed the study design, data collection, and mastered the data. SJ, AA, RG, and GP ran the data analysis. ER, GL, and GP discussed the results. VP and AA wrote the manuscript with the support of RG, GP, and ER. SJ, ER, and GL supervised the project and manuscript preparation. All authors contributed to the article and approved the submitted version.

## Conflict of Interest

The authors declare that the research was conducted in the absence of any commercial or financial relationships that could be construed as a potential conflict of interest.
