# Supplementary material for: Do Social Connections and Digital Technologies Act as Social Cure During COVID-19?
Source: Front Psychol. 2021 Apr 1;12:634621. doi: 10.3389/fpsyg.2021.634621 (PMC8047103; doi:10.3389/fpsyg.2021.634621)
Supplement: Supplementary file 1 [file Data_Sheet_1.PDF]

Themes and Sub-themes extracted from the responses of the Patients and Care-givers

Themes	Subthemes
Psychological Experiences of People	Perceived experiences
	Concern towards patients
	Negligence of psychological counselling
Attitude of others toward the Patients & Caregivers	During Recovery
	After Recovery
Digital Technology	Influence of Digital Media
	Technology Aids
Social Connectedness	Social connection and Social Media
	Preventive Measures
	Altruistic Volunteers

(Source: Prepared by Authors)
